# „Frauen, Frieden und Sicherheit“ unter den Bedingungen der COVID-19 Pandemie

**DOI:** 10.1007/s42597-020-00045-x

**Published:** 2020-10-30

**Authors:** Manuela Scheuermann

**Affiliations:** Institut für Politikwissenschaft und Soziologie, Julius-Maximilians-Universität Würzburg, Wittelsbacherplatz 1, 97074 Würzburg, Deutschland

**Keywords:** COVID-19 Pandemie, Geschlechterstereotype, Rollenbilder von Frauen, Feministische Friedensforschung, Partizipation von Frauen, Globale Fürsorge, Gender stereotypes, Female roles, Feminist peace research, Female participation, Global care

## Abstract

Die COVID-19 Pandemie und ihre gesamtgesellschaftlichen Folgen werden zum Stresstest für die globale Agenda „Frauen, Frieden und Sicherheit“. Mit dieser Agenda verfolgt die Weltgemeinschaft seit dem Jahr 2000 das Ziel, Frauen in Situationen von gewaltsamen Konflikten und in der Phase des Wiederaufbaus vor Gewalt und Menschenrechtsverletzungen zu schützen, ihnen eine belangvolle Partizipation im Friedensprozess zu ermöglichen und so zu einem gendersensiblen Friedensbildungsprozess beizutragen. Im vorliegenden Beitrag wird argumentiert, dass die Folgen der Corona-Krise einen Rückfall im Implementierungsprozess der Agenda auslösen könnten, insbesondere in Bezug auf Geschlechterstereotype. Es wird diskutiert ob bestimmte als traditionell-weiblich perzipierte Rollen verfestigt werden und welche Auswirkungen diese Beobachtung auf die Zukunft der globalen Agenda haben könnte. Von besonderer Bedeutung ist hierbei das Konzept der globalen Fürsorge.

## Abstract (en)

The COVID-19 pandemic and its consequences for society as a whole are becoming a stress test for the global agenda “Women, Peace and Security“. With this agenda, the international community has been pursuing since 2000 the goal of protecting women in situations of violent conflict and in the reconstruction phase from violence and human rights violations, enabling them to participate in the peace process in a meaningful way and thus contributing to a gender-sensitive peacebuilding process. The paper argues that the consequences of the Corona crisis could trigger a relapse in the implementation process of the Agenda, especially with regard to gender stereotypes. It is discussed whether certain roles perceived as traditional feminine are being consolidated and what effects this observation could have on the future of the global agenda. The concept of global care is of particular importance.

## Einleitung

Rund ein halbes Jahr nach Ausbruch der COVID-19 Pandemie zeichnet sich ab, dass die Pandemie und ihre gesamtgesellschaftlichen Folgen gewaltsame Konflikte katalysieren, gesellschaftlichen Unfrieden stiften und zugleich internationale Maßnahmen zur Friedenssicherung schwächen.^1^ Diese Situation, die in vielen Konfliktstaaten zu beobachten ist, könnte sich nach Ansicht der „Group of Women Leaders“ zu einer weiblichen Krise innerhalb der Krise entwickeln (2020). Pandemiebedingt könnten die Bemühungen um Geschlechtergleichberechtigung und -gerechtigkeit auf lokaler und schließlich auch auf globaler Ebene ausgebremst werden. Das betrifft vor allem die internationalen Bemühungen rund um die globale Agenda für „Frauen, Frieden und Sicherheit“ (FFS). Zugleich stellen Klugman und Haiewen Zoe vom „Women, Peace and Security“-Index fest, dass Staaten, in denen die Lebenssituation von Frauen bereits vor der Pandemie als „desaströs“ eingestuft wurde, durch die Folgen der Pandemie besonders getroffen sind (2020).

Der vorliegende Beitrag nimmt diese Entwicklungen zum Anlass, um die Folgen der Pandemie vor der Hintergrundfolie der globalen Agenda für „Frauen, Frieden und Sicherheit“ und mit dem Fokus auf Geschlechterstereotype, auch Geschlechterrollen genannt, zu diskutieren. Damit trägt der Beitrag der Erkenntnis Rechnung, dass Geschlechterrollen insbesondere in Krisenzeiten einem Anpassungs- oder Veränderungsdruck ausgesetzt sind. Es wird argumentiert, dass die COVID-19 Pandemie in Konfliktstaaten unmittelbare Auswirkungen, sogenannte „gendered effects“ (Makan-Lakha und Hamilton 2020), auf die Geschlechterrollen vor Ort hat. Verfestigen sich diese Effekte, so könnten sich durch die Pandemie und ihre Folgen Geschlechterstereotype auch längerfristig und über die lokale Ebene hinaus verändern. Sie könnten mittel- und langfristig die globale Agenda für „Frauen, Frieden und Sicherheit“ negativ beeinflussen. Denn letztlich ist es doch der normative Kern von FFS, Geschlechterrollen im feministischen Sinne zu verändern und für Geschlechtergerechtigkeit zu sorgen, gerade in Konflikt- und Post-Konfliktstaaten. Eine Renaissance traditioneller Geschlechterrollen könnte den „backlash“, dem die Agenda aufgrund der aktuellen Nationalismen, antifeministischen Strömungen und unilateralistischen Tendenzen insbesondere im UN-Sicherheitsrat (SR) ausgesetzt ist, weiter verstärken.

Um diese möglichen „gendered effects“ der Pandemie feststellen und bewerten zu können sowie Implikationen für die FFS-Agenda abzuleiten, werden Daten aufbereitet, die vor allem UN Women in Zusammenarbeit mit der Weltgesundheitsorganisation seit Ausbruch der Pandemie gesammelt hat (UN Women 2020a, 2020b, 2020c, 2020d, 2020e). Diese beiden Organisationen sind die einzigen Akteure, die derzeit systematisch und umfangreich entsprechende geschlechtsspezifische Daten auswerten. Deren Ziel ist es, eine „Gender Brille“ in den Diskurs um die Folgen der Pandemie einzubringen. Das „Centre for Feminist Foreign Policy“ hat eine umfangreiche Bibliografie zu feministischer Literatur in Bezug auf die Pandemie erstellt. Im „Georgetown Institute for Women, Peace and Security“ und „LSE Centre for Women, Peace and Security“ wird der Diskurs auf hochrangiger wissenschaftlicher und politischer Ebene geführt. Auch deren Erkenntnisse fließen in den vorliegenden Beitrag ein. Alle bisherigen Veröffentlichungen sind eher die Spiegelung von Momentaufnahmen. Sie sind der leise Versuch, aus den bereits beobachtbaren und sich verfestigenden Entwicklungen Implikationen für die Zukunft der Agenda abzuleiten.

Im Folgenden werden das Konzept der Geschlechterstereotype in aller Kürze erläutert und kontextspezifische Rollen vorgestellt. Danach wird die globale Agenda für Frauen, Frieden und Sicherheit eingeführt, um die aktuellen Entwicklungen vor der Hintergrundfolie der Agenda und mit dem Ziel, Veränderungen weiblicher Geschlechterstereotype abzuleiten, zu diskutieren. Zuletzt werden diese Beobachtungen auf die globale Ebene der Agenda für „Frauen, Frieden und Sicherheit“ transferiert.

## Frieden, Sicherheit und Geschlechterstereotype

Geschlechterstereotype „sind kognitive Strukturen, die sozial geteiltes Wissen über die charakteristischen Merkmale von Frauen und Männern enthalten“ und einen prä- sowie einen deskriptiven Anteil haben (Eckes 2008, S. 178). Während die deskriptiven Annahmen die Realität der Geschlechterrollen abbilden, beispielsweise die traditionelle These, dass Frauen fürsorglicher und Männer dominant sind, führt die präskriptive Annahme in den Bereich des Sollens.

Die feministische Friedensforschung leitet die Geschlechterstereotype aus der Analyse traditioneller Konzepte von Frieden und Sicherheit ab. Nach Tickner (2019) wird Sicherheit in der herkömmlichen Auffassung mit starker militärischer Maskulinität assoziiert, während Frieden mit schutzloser Weiblichkeit, der Frau als dem Opfer von Unsicherheit, verbunden wird. Feministische Forschung stellt die oftmals als natürlich-frauenspezifisch perzipierte essentialistische Verbindung von „Frau und Frieden“ oder „weiblich ist gleich friedlich“ oder „weiblich ist gleich schutzbedürftig“ ebenso in Frage wie die Verbindung von „männlich und militaristisch“, „männlich und schützend“ oder „männlich und verteidigend“. Sie fordert damit die deskriptiven traditionellen Geschlechterstereotype heraus und setzt der These der friedvollen Frau die präskriptive Auffassung entgegen, dass Frauen Akteure aller Facetten von Friedensstiftung („peacemaking“) seien sollen (Tickner 2019). Diese präskriptiven Stereotype, die aus feministischer Perspektive auch der globalen Agenda für „Frauen, Frieden und Sicherheit“ zugrunde liegen, verfolgen eher einen Ansatz der Gender-Neutralität.

Derzeit scheint es so, als ob genau dieser Ansatz durch die Realitäten der Pandemie herausgefordert würde. In vielen Industriegesellschaften wird der Rückfall in die patriarchale Rollenverteilung der 1950er-Jahre prognostiziert (Allmendinger 2020). Für Konfliktstaaten könnte gelten, dass Frauen als „Corona-Opfer“ in die Rolle von „Kriegsopfern“ zurückfallen. Frauen könnten zudem aus der öffentlichen in die private Sphäre der Gesellschaft zurückgedrängt werden. Dieser Rückfall ins Private nähme ihnen zugleich die Möglichkeit, als gleichberechtigte gesellschaftspolitische Akteur*innen beim Wiederaufbau des jeweiligen Landes mitzuwirken und so Geschlechterrollen dauerhaft aufzuweichen, ja zu verändern. Das würde auch die globale Agenda fundamental schwächen.

## „Frauen, Frieden und Sicherheit“ in Zeiten der Pandemie

Die globale Agenda „Frauen, Frieden und Sicherheit“ ist ein vielschichtiges und vielgestaltiges Projekt, das inzwischen zehn Resolutionen des UN-Sicherheitsrats (SR) umfasst.^2^ Derzeit überwiegt die Ansicht im SR, das normative Fundament dieser zehn Resolutionen sei ausreichend, um alle Facetten des Themas abzudecken (Security Council Report 2020). Die Staatengemeinschaft müsse sich stärker auf die Implementation konzentrieren (S/RES/2493). Diese Entwicklung mag in den normativen Grabenkämpfen begründet sein, die sich 2019 im SR auch und gerade bei Fragen der Gleichberechtigung der Geschlechter, der feministischen oder antifeministischen Agenda mancher SR-Mitglieder und konkreten Fragen beispielsweise zu „reproductive health“ abspielten (Security Council Report 2020). Die Resolutionen zu FFS bildeten schon immer den Minimalkonsens zwischen der einflussreichen feministischen Zivilgesellschaft und den Konservativen unter den SR-Mitgliedern ab. Fördert die überwiegende Mehrheit der Staaten Geschlechterparität um der Geschlechterparität willen, treten feministische Regierungen wie Schweden für Frauen am Konferenztisch ein, um das patriarchale und maskulinisierte System grundlegend verändern zu können. Der Agenda liegt demnach die feministische Idee zugrunde, dass ein weiblicher Blick auf Konfliktlösung und Friedensbildung die normativen Grundlagen und damit auch das Instrumentarium der Friedenssicherung und des Wiederaufbaus grundlegend verändern würde. Auch die in den UN-Gremien einflussreichen FFS-Gruppen, beispielsweise die „Women’s International League for Peace and Freedom“, zielen nicht nur darauf ab, die Akteursqualität der Frauen zu stärken, Frauen zu „empowern“ oder die Anzahl von Frauen in Führungspositionen zu erhöhen. Mit Hilfe der Agenda sollen Machtverhältnisse und Geschlechterrollen verändert, also eine feministische Wende im Bereich Frieden und Sicherheit eingeleitet werden.

Einig sind sich alle Akteure in den vier Schwerpunkten der Agenda. Sie kann in die Säulen „Schutz“, „Beteiligung“, „Prävention“ sowie „Nothilfe und Wiederaufbau“ unterteilt werden. Die Säule des Schutzes thematisiert den Schutz der Frau vor jeglicher Form von psychischer und physischer, insbesondere sexueller und gender-basierter Gewalt, und die Anerkennung von Frauenrechten als Menschenrechte. Aus dieser in vielen Resolutionen stark betonten Schutz-Komponente folgt, dass Frauen im Kontext von FFS oftmals als „Opfer“ und „Schutzbedürftige“ perzipiert werden. Mit Resolution 2467 (2019) wurde die Rolle der „Überlebenden“, also eine weit pro-aktivere Rolle, eingeführt, um den traditionellen weiblichen Stereotyp der Hilf- und Schutzlosen abzuschwächen. Die Säule der Beteiligung subsummiert alle Formen der belangvollen weiblichen Teilhabe an der Lösung des Konflikts und dem Wiederaufbau des Landes, von der lokalen „first responder“ bis zur Kommandeurin einer UN-Friedensmission. Im Kern bemüht diese Säule die gleichberechtigte Führungsrolle von Frauen in allen Bereichen von Krieg und Frieden. Es ist die Säule, die letztlich die hegemoniale militarisierte Männlichkeit, die in vielen Konflikten zu beobachten ist und mit den männlichen Rollen der Verrohung, der Dominanz und Gewalt verbunden ist, herausfordern könnte (Messerschmidt und Quest 2020). Die Kultur der Konfliktprävention beruht auf der Prämisse, dass Geschlechtergerechtigkeit Frieden fördert. Frieden ist im Kontext der Agenda in einen umfassenden und menschlichen Sicherheitsbegriff eingebettet, der Trias „freedom from want, freedom from fear and freedom to live in dignity“ (UNDP 2013). Die Agenda geht davon aus, dass die Zukunft dann geschlechtergerechter wird, wenn in Nothilfe und Wiederaufbau gendersensitive Maßnahmen einfließen. Auch diese Säule hebt auf die feministische Idee ab, dass Frauen alle Rollen annehmen können, die Wiederaufbausituationen erfordern.

Eine weitere, vom UN-Sicherheitsrat mitgetragene Entwicklung, die zumindest eine Durchlöcherung oder Aufweichung von Rollen befördern könnte, ist die Integration des intersektionalen Ansatzes in die FFS-Resolutionen (Zürn 2020). Der Terminus der Intersektionalität bildet ein Bündel theoretischer Ansätze ab, die sich auf das Wechselverhältnis von Geschlecht und weiteren sozialen Ungleichheiten beziehen (Lenz 2010, S. 158). Die Begriffsschöpferin Crenshaw begründet die Relevanz wie folgt: „Because of their intersectional identity as both women and people of color within discourses that are shaped to respond to one or the other, the interests and experiences of women of color are frequently marginalized within both.“ (1991, S. 1242) Es muss also stets mitbedacht werden, dass sich Geschlechterstereotype intersektional ausbilden. Das gilt insbesondere in Konfliktstaaten, in denen wir diverse interdependente Ungleichheiten beobachten, die durch die Pandemie und ihre Folgen noch verstärkt werden könnten.

### Schutz: Die Rolle des Opfers und der Rückzug ins Private

Vor dem Hintergrund der COVID-19 Pandemie muss der Aspekt der Schutzbedürftigkeit, der mit dem sehr traditionellen Geschlechtsstereotyp der friedvollen, zu schützenden Frau in Verbindung steht, besonders betrachtet werden.

Drei der Pandemie geschuldete Entwicklungen sind im Bereich der Schutz-Säule von FFS zu beobachten:

1. die Zunahme von geschlechtsspezifischer Gewalt,
2. die Zunahme der Müttersterblichkeit,
3. die nachlassende Anerkennung von Frauenrechten als Menschenrechten.

Besonders signifikant ist der weltweit zu beobachtende, steile Anstieg von häuslicher Gewalt, die sich doch bereits vor der Pandemie auf einem „epidemischen Level“ befand (Guterres 2020a). Bleiben die Fallzahlen längerfristig auf einem hohen Niveau, kann Gewalt gegen Frauen durchaus das gesamtgesellschaftliche Gefüge destabilisieren und zu Unfrieden führen. In Anlehnung an Zwingel führt Harders dazu aus:

Wenn also häusliche Gewalt und andere Formen sexualisierter Gewalt von Staaten nicht als elementares Sicherheits- und Demokratieproblem wahrgenommen werden, dann kann das Ausmaß privatisierter geschlechtsspezifischer Gewalt dazu führen, dass auch in Friedenszeiten Unfrieden den Alltag von Frauen prägt. (…) Diese Formen der nur scheinbar privaten Gewalt in Friedenszeiten sind eng mit den geschlechtsspezifischen Gewaltformen des Krieges verbunden. (Harders 2010, S. 533)

Dies gilt umso mehr in Konfliktländern. In wohlhabenden Industrienationen wie Frankreich stieg die Rate gemeldeter (!) Fälle von geschlechtsspezifischer Gewalt im Lockdown um 30 %, wohingegen die Dunkelziffer schon vor der Pandemie um 60 % höher lag (Miliband 2020). In Nigeria wird die Hotline gegen sexuellen Missbrauch dreimal so häufig frequentiert wie vor COVID-19. Die Gewalt trifft dort besonders junge Mädchen (Thielke 2020). Das kann Teenie-Schwangerschaften unter prekärsten Bedingungen zur Folge haben. Dies war bereits während des Ebola-Ausbruchs in Sierra Leone 2014 zu beobachten (Miliband 2020). UN Women reagiert auf den Anstieg der Gewalt mit der Kampagne „Shadow Pandemic“ (UN Women 2020e). Die Organisation sensibilisiert vor allem für Folgen dieser Gewalt für das gesamtgesellschaftliche Friedensprojekt. Laut UN Women führen wirtschaftlicher und sozialer Stress in Verbindung mit Bewegungseinschränkungen und beengten Wohnverhältnissen zu mehr geschlechtsspezifischer Gewalt (UN Women 2020a, 2020b). Während Frauen in den Industriestaaten unbezahlten Urlaub einreichen oder im Homeoffice arbeiten, um die Folgen der Pandemie, insbesondere den Wegfall von Kinderbetreuung und Schule, abzufedern, haben Frauen in Konfliktländern ihre prekäre Beschäftigung mit dem Lockdown verloren. Die Pandemie-bedingte oder -verschärfte finanzielle Unmündigkeit treibt Frauen in die private Abhängigkeit vom Ehemann, mit allen oft gewaltsamen Konsequenzen. Guterres konstatierte bereits am 29. April dieses Jahres, dass die wirtschaftliche Krise ein weibliches Gesicht haben werde (Guterres 2020c). Große wirtschaftliche Unsicherheit, soziale Isolation und der Verlust der Arbeit binden Frauen an zu Hause und an die Gewalttäter. UN Women befürchtet in diesem Zusammenhang auch mehr Femizide (UN Women Europe and Central Asia 2020). Notunterkünfte wie Frauenhäuser sind geschlossen, Gesundheitszentren zur Behandlung der Frauen und Justizsysteme zur Ahndung der Taten überlastet. Humanitäre Organisationen fallen als Ansprechpartner vor Ort oftmals aus. Sie sind selbst vom Lockdown betroffen, ziehen ihre Mitarbeiter*innen ab und fürchten aufgrund der weltwirtschaftlichen Situation nach der Pandemie um ihre Existenz (Bhatia 2020). Frauen sind einem erheblichen Gewaltrisiko ausgesetzt, wenn sie sich auf durch den Lockdown verursachten menschenleeren, aber stärker militarisierten Straßen bewegen. Der Gang zur Latrine, zum Brunnen oder zu Wasserverteilungsstellen, eine „typisch weibliche“ Aufgabe, kann ein weiteres Einfallstor für geschlechtsspezifische Gewalt sein. Das Programm „Sicher und fair“ im asiatisch-pazifischen Raum berichtet über ein erhöhtes Risiko sexueller Ausbeutung und Gewalt durch die Polizei und bewaffnete Wachen bei Grenzkontrollen. Zudem belegt UN Women ein erhöhtes Risiko psychologischer Gewalt für Wanderarbeiterinnen, die ihren Arbeitsplatz verloren haben und nicht mehr in der Lage sind, ihre Familien zu ernähren. Die Wahrscheinlichkeit von Mädchenheirat und sexueller Ausbeutung könnte ebenfalls zunehmen, um existentielle Notlagen abzufedern. Für die Rolle von Frauen im Kontext von FFS bedeutet dies einen fundamentalen Rückfall in die Opferrolle, in die Rolle einer hilfsbedürftigen, schutzsuchenden und zugleich hilf- und schutzlosen Frau. Es ist ein unfreiwilliger Rückfall in traditionelle Stereotype der privaten, erwerbslosen und vom Mann abhängigen Hausfrau. Dem steht der präskriptive Typus einer selbstbewussten und eigenständigen Gestalterin der Zukunft doch fundamental entgegen.

Bereits vor der Pandemie war die Rate von Müttern, die bei oder nach der Geburt ihres Kindes versterben, mit 300 Todesfällen bei 100.000 Lebensgeburten in Konfliktländern sehr hoch. Die höchste Rate verzeichnete Südsudan mit 1150 Müttern bei 100.000 Geburten, in Sierra-Leone starben 1120 Mütter pro 100.000 Geburten (WHO et al. 2019). Der Ebola-Ausbruch in Westafrika, der den Gesundheitssektor stark belastete, führte zu einem extremen Anstieg der Müttersterblichkeit. In Sierra Leone lag die Mütter- und Säuglingssterblichkeit so hoch wie die Sterblichkeit durch Ebola, nämlich bei „3600 additional maternal, neonatal and stillbirths deaths in the year 2014–15“ (Sochas et al. 2017, S. 23). Diese Situation könnte sich gegenwärtig wiederholen. Roberton et al. (2020) schätzen, dass zusätzlich bis zu 56.700 Frauen während oder nach der Geburt unter Corona-Bedingungen sterben könnten. Wichtige prä- und postnatale Kontrolluntersuchungen werden in vielen instabilen Regionen derzeit nicht durchgeführt. Schon einfachste Hygienestandards für schwangere, gebärende oder menstruierende Frauen fehlen. Ob die von Deutschland erfolglos gepushte Inklusion von „reproductive health“ in Resolution 2467 (2019) des UN-Sicherheitsrats die Situation abgefedert hätte, kann an dieser Stelle nicht diskutiert werden. Fakt ist jedoch, dass Frauen derzeit aufgrund medizinischer Notlagen noch stärker auf sich selbst zurückgeworfen, nicht wahrgenommen und/oder diskriminiert werden.

In dieser Gemengelage ist der Schutz von Frauenrechten in Gefahr, zumal der folgende Abschnitt zeigen wird, dass Frauen aufgrund der mangelnden politischen Gleichberechtigung ihre Rechte nicht verteidigen können. Zwar lernen auch sie aus der Krise, sie haben aber wenige Möglichkeiten, das gemeinsamen Projekt „build back better“ (United Nations 2020, S. 2) an den Schaltstellen der Welt mitzugestalten. Es ist eine frauenrechtlich fatale Wirkung der Pandemie, dass Frauen aufgrund der geschilderten Situation von Frauennetzwerken, „grassroot“-Aktivitäten und öffentlichen Beteiligungsmöglichkeiten abgeschnitten sind.

### Teilhabe und Konfliktprävention: Frauen spielen nur selten eine Rolle

Die derzeit starke Schutzbedürftigkeit vieler Frauen in Konfliktländern lässt die Frage nach der Teilhabe als gleichberechtigte Akteurin in der Konfliktprävention, der Friedenssicherung und beim Wiederaufbau fast absurd erscheinen. Ein Blick auf die Situation vor Ausbruch von COVID-19 und in die Corona-Task-Forces der Welt kann jedoch Hinweise auf die Zukunft dieser Säule und der damit verbundenen Rollen geben.

Bereits vor der Pandemie waren Frauen nur selten ein integraler und offizieller Teil der Konfliktlösung. Auch wenn bereits seit dem Jahr 2000 die stärkere Teilhabe von Frauen in allen Bereichen von Konfliktlösung, Prävention und Wiederaufbau angemahnt wird, sind nur drei Prozent der Konfliktmediator*innen, 13 % der „peace negotiators“ und 4 % der UN-Blauhelme weiblich (Council on Foreign Relations 2019). Weiblicher Einfluss findet derzeit fast ausschließlich auf dem zivilgesellschaftlichen „grassroot“-Level statt. Bei lokalen Konfliktsituationen oder Epidemien wie dem Ebola-Ausbruch 2019 in der Demokratischen Republik Kongo sind Frauen die unverzichtbaren „first responder“ (WHO 2019). Wenn Frauen jedoch nicht am Konferenztisch sitzen, finden frauenpolitische Anliegen zumeist keinen Eingang in die Friedensverhandlungen. Nur 19,7 % aller zwischen 2000 und 2018 unterzeichneten Friedensabkommen waren gendersensibel angelegt (UN Security Council 2019, § 15). Vielen Friedensaktivist*innen ist in der Corona-Pandemie das politische Netzwerken unmöglich geworden. Ihnen fehlen auch die finanziellen Grundlagen für die feministische Friedensarbeit.

Die Praxis, Frauen bei Entscheidungen von gewaltiger Tragweite für die Zukunft eines Staates außen vor zu lassen, ist auf die aktuelle Situation vollständig übertragbar. Augenfällig waren zu Beginn der Pandemie die männlich dominierten Corona-Task-Forces oder Expertengremien, beispielsweise in Frankreich, Italien, Deutschland und den USA, aber auch in der Weltgesundheitsorganisation und „WHO-China joint mission on COVID-19“. Die vielen genderspezifischen Folgen von männlich dominierten Task-Forces, die hier nicht behandelt werden können, diskutiert Felton (2020). Auch wenn Länder wie Italien ihre Task-Forces nach öffentlicher Kritik weiblicher besetzten (Abramo 2020), wurde der Forderung nach mehr weiblicher Teilhabe entgegengehalten: „We’re in a wartime, and we have to prioritze what’s urgent“ (Gharib 2020). Männliches Storytelling mittels maskulin perzipierter Kampf-Begriffe führt nicht selten zu männlichen Maßnahmen und dem Ausschluss der „friedvollen“ Frauen.

Die mangelnde Geschlechterparität in politischen Leitungsfunktionen könnte auch in der COVID-19 Pandemie dazu führen, dass frauenpolitische Belange nicht auf die Agenda gelangen. Beispielsweise sind nur 24,7 % der Gesundheitsminister*innen weltweit Frauen (UN Women 2020a). Sie verantworten die Maßnahmen gegen die Pandemie ebenso wie die 6,2 % weiblichen Regierungschef*innen. 13,2 % weibliche Finanzminister*innen befinden über die Verteilung der Finanzmittel in der größten Wirtschaftskrise seit dem Zweiten Weltkrieg (UN Women 2020a). Grundsätzliche und altbekannte Probleme werden nun unter den Bedingungen dieser Krise virulent und könnten dazu führen, dass Maßnahmen wie beispielsweise stimulierende Anreize des IMF eben nicht frauenbezogen sind (Bhatia 2020). Gesellschaftlicher Unfrieden „unten“ erwächst auch aus mangelnder gleichberechtigter Teilhabe „oben“.

Was bedeutet dies nun für Geschlechterstereotype in Bezug auf die globale Agenda? Alte Geschlechtermuster, die durch die zweite Säule von FFS aufgebrochen werden sollen, könnten durch die derzeitige Situation verfestigt werden – weil Männer führen, weil Frauen ins Private zurückgedrängt werden, weil Frauen derzeit keine Anreize, keine Mittel und schlicht keine Zeit haben, selbst „Actorness“ zu zeigen. Die Rolle der Frau als Krisenmanagerin könnte sich im Portfolio der präskriptiven Rollenbilder merklich abschwächen.

### Eine neue Rolle für FFS: Die Fürsorgende

Ein ganz anderer Kontrapunkt, den dieser Beitrag in Bezug auf Geschlechterstereotype im Bereich von FFS setzen möchte, verweist auf eine sehr traditionelle weibliche Rolle – den deskriptiven Typus der Fürsorgenden und Pflegenden. Im Folgenden soll kurz theoretisch über Fürsorge im globalen Kontext nachgedacht werden, um dann die empirische Situation während der Corona-Pandemie zu schildern. Es stellt sich die Frage, ob die Pandemie zu einer veränderten Konnotation dieses Geschlechtsstereotyps beitragen und was dies wiederum für FFS bedeuten könnte.

1997 konzeptualisierte Robinson unter Rückgriff auf die feministischen Arbeiten zu Care-Ethik von Gilligan (1982) die „Fürsorge im globalen Kontext“ und die „morality of care“. Auch wenn die Pandemie per se kein moralisches Problem ist, wirft sie doch viele grundsätzliche moralische Fragen auf – gerade im Fürsorge-Sektor (Gabriel 2020). Gilligan beobachtete, dass Frauen und Männer unterschiedliche Ansätze wählen, um mit moralischen Fragen, Problemen und der Praxis von Fürsorge umzugehen. Sie erkannte, dass Frauen in moralischen Konflikten anders entscheiden als Männer. Frauen bewerten die moralische Frage nach Fürsorge-ethischen Aspekten, wohingegen Männer Rechte-geleitet urteilen (Gilligan 1982). Der moralische Kern von Fürsorge, so Robinson, setzt jedoch eben nicht Rechte-geleitetes Handeln aus Eigeninteresse voraus, sondern konstituiert sich aus interpersoneller Aufmerksamkeit, Verantwortungsbewusstsein und sozialer Responsivität mit einem Gespür für Praktikabilität und Kontext (Robinson 1997, S. 121). Das sind die typischen Merkmale eines traditionellen weiblichen Geschlechterstereotyps. Robinson forderte 1997, dass sich diese als typisch weiblich perzipierte Fürsorgemoral nicht auf den privaten Bereich beschränken solle, sondern auf globaler Ebene gelten müsse (Robinson 1997).

In der Corona-Krise scheint diese Forderung zumindest von den weiblichen Staats- und Regierungschefinnen umgesetzt zu werden. Aktuell medial weit verbreitete Nachrichten charakterisieren deren Krisenmanagement mittels eben jener Tugenden, die dem Robinsonschen Global Care-Konzepts entlehnt scheinen (Wittenberg-Cox 2020).^6^ Den Regierungschefinnen werden vor allem Menschlichkeit, Fürsorge, Praktikabilität und Verantwortungsbewusstsein attestiert. Feminist*innen wie Lewis kritisieren diese Zuschreibungen. Sie argumentieren, dass diese traditionellen geschlechterstereotypen Beschreibungen destruktiv auf das feministische Projekt wirken (Lewis 2020). Letztlich könnten Geschlechterstereotype verfestigt und fortgeschrieben werden, die doch zu überwinden sind (True und Davies 2020). True und Davies (2020) befürchten sogar einen „backlash [for] (…) female leaders“. Aber könnte eine größere Anerkennung globaler Fürsorge in Corona-Zeiten nicht auch dazu führen, dass Fürsorge dauerhaft positiver konnotiert wird? Könnte es nicht auch dazu beitragen, dass positiv gedeutete traditionelle Geschlechterstereotype zu mehr Akteursqualität von Frauen beitragen, diese dazu ermutigen, eine gleichberechtigte, aber doch andere, neu bewertete traditionelle Rolle zu spielen? Könnte eine positivere Konnotation traditioneller Rollenbilder vielleicht sogar den Backlash, den FFS derzeit erlebt, etwas abfedern?

Der von Frauen dominierte Fürsorge-Sektor kann für weitere Überlegungen ein erster Ansatzpunkt sein. Er zeigt, dass eine positive Umdeutung dieser traditionellen Frauenrolle eine sehr große Anzahl von Frauen ermächtigen könnte.

Fakt ist nämlich: die Parität der Geschlechter kehrt sich im Fürsorge-Sektor um, sowohl im Bereich der bezahlten als auch der unbezahlten Arbeit. In der Pandemie wird diese Situation verstärkt wahrgenommen. Die globale Arbeitskraft in der Fürsorge und Pflege ist hochgradig verweiblicht. 70 % der im Fürsorge-Bereich Beschäftigten sind Frauen (Boniol et al. 2019). Auch in der unbezahlten Fürsorge- und Hausarbeit waren bereits vor der Pandemie dreimal so viele Frauen engagiert wie Männer (UN Women 2019).^7^ Die gesamte unbezahlte Arbeit von Frauen, insgesamt schätzungsweise 12,5 Mio. Stunden, kommt einem Geldwert von 10,8 Billionen US-Dollar pro Jahr gleich (Oxfam 2020). Vor allem Frauen kümmern sich in der Pandemie um die grundlegenden Überlebensbedürfnisse der gesamten, zumeist sehr großen Familie. Dies umso mehr, wenn Gesundheitssysteme zusammenbrechen und Kranke zu Hause versorgt werden müssen. Frauen werden in ihre Rolle als Fürsorgende, das Praktische Organisierende, die für das Wohl der Familie Verantwortliche, die Pflegende und Verstehende zurückgeworfen.

Auf den ersten Blick spricht diese Situation für einen extremen Rückfall in alte Rollenbilder und für einen Abgesang gleichberechtigter weiblicher Teilhabe im Bereich von Frieden und Sicherheit. So wurde in den vorherigen Kapiteln letztendlich auch argumentiert, wenn es um die Rolle als Opfer oder Nicht-Teilhabende ging. Folgt man dieser Sichtweise, so muss konstatiert werden, dass die derzeitige Lebenswirklichkeit fatale Auswirkungen auf das gesamte Projekt der globalen Agenda hat. Und dies auch in Zukunft, denn Kinder lernen derzeit ein Geschlechterverhältnis und eine weibliche Rolle kennen, die als längst überholt galt. Die Ungleichheit der Geschlechter, insbesondere in den armen Milieus, könnte sich in die nächste Generation einprägen. In einer Rede vom 9. April 2020, in der Guterres über Ungerechtigkeit als „Wesensmerkmal(s) unserer Zeit“ (Guterres 2020a) sprach, benannte er auch die Ursache, die diese nun wieder dominante weibliche Rolle verfestigen könnte, nämlich „das seit Jahrtausenden bestehende Patriarchat. Wir leben in einer männlich dominierten Welt mit einer männlich dominierten Kultur. Überall sind Frauen schlechter gestellt als Männer, aus dem einfachen Grund, dass sie Frauen sind“ (Guterres 2020b).

Allerdings könnte diese Situation des Rückfalls in die Fürsorge bei gleichzeitiger Erkenntnis, dass Fürsorge von Frauen dominiert ist, eine Chance für eine veränderte Konnotation von Fürsorge sein. Sie könnte dafür sorgen, dass weibliche Attribute der „morality of care“ in männlichen Kontexten von Krieg und Sicherheit nicht mehr als per se weich und schwach wahrgenommen werden. In der aktuellen Pandemie wird sehr deutlich, dass bezahlte und unbezahlte Fürsorge die Grundlagen der menschlichen Existenz und des gesellschaftlichen Friedens sind. Fürsorgende, also die Frauen, sichern aktuell das grundlegende Überleben.

Deshalb unternimmt UN Women bereits Anstrengungen, die Fürsorge-Arbeit insgesamt positiv zu triggern. Sollte diese Strategie beibehalten werden, müsste sich das Department of Peace Operations der Vereinten Nationen auch nicht länger darum bemühen, weibliche Blauhelm-Soldatinnen in möglichst maskulinen und militarisierten Posen zu zeigen, sondern als die fürsorgenden und sozial responsiven Kommunikator*innen, als die sie in UN-Einsätzen geschätzt werden. Eine positivere Perzeption dieser Fürsorge-Rolle, die unbestritten als ein weibliches Attribut gilt, könnte die Agenda auf eine andere, vielleicht auch realitätsnähere normative Basis stellen. Sie könnte Frauen ermutigen, diesen Teil ihrer weiblichen Identität als ihre Stärke zu begreifen und sie bestärken, auch in als männlich wahrgenommen Bereich von Frieden und Sicherheit als moralisch-fürsorgende Akteur*innen in Erscheinung zu treten. Umgekehrt gilt: auch wenn eine positivere Interpretation dieser Geschlechterrolle die Aussicht auf Wandel durch Bewusstwerden in sich trägt und definitiv das Potenzial hat, das globale Miteinander zu verändern, darf ein starker Fokus der Frauen auf Fürsorgearbeit nicht dazu führen, dass sie nur noch Fürsorge leisten. Eine Person, die nur im Privaten agiert, ist dort schutzlos und hat kaum Möglichkeiten, an der Gestaltung der Gesellschaft mitzuwirken. Die Aufwertung des Fürsorge-Attributs kann nur gelingen, wenn Fürsorge zu einer globalen Norm wird.

## Die COVID-19 Pandemie und ihre Folgen für die globale FFS-Agenda

Für die globale Agenda bedeuten diese Entwicklungen, sich nochmals normativ mit offenkundig starken weiblichen Stereotypen und deren Potenzial für die Friedensstiftung auseinanderzusetzen. Dazu muss ein ganz spezifischer Resilienzgedanke stärker verankert werden, der Frauen davor schützt, in Krisen wie der aktuellen in Rollen gedrängt zu werden, die sie schutzlos und mitunter auch rechtlos zurücklassen. Gleichzeitig muss zumindest die Bereitschaft vorhanden sein, den Wert des Fürsorge-Konzepts für die globale Agenda zu diskutieren. Gleichzeitig muss die weibliche belangvolle und einflussreiche Teilhabe an politischen Prozessen vorangebracht werden (United Nations Peacekeeping 2020). Dazu ist ein gesamtgesellschaftliches Umdenken nötig, das langfristig zu einem geschlechtergerechten Wandel führen könnte. Ob dieser normative Bewusstseinswandel angesichts des globalen Machtgefüges im SR derzeit realistisch ist, sei dahingestellt. Um so stärker liegt es in den Händen der Zivilgesellschaft, die internationale Gemeinschaft davon abzuhalten, nach der Aufbereitung der doch frustrierenden Daten in ein „business as usual“ zu verfallen. Es ist das Gebot der Stunde, aus den derzeitigen Erfahrungen zu lernen und die Chance des „build back better“ zu ergreifen (Verveer in Lieberman 2020).

## Footnotes

1 De Coning (2020), Di Razza (2020), Gowan und Andersen (2020), Mustasilta (2020), Okeke (2020), Sciorati (2020). Dagegen argumentiert Posen (2020), die Pandemie würde das Risiko eines Krieges reduzieren.

2 S/RES/1325 (31. Oktober 2000), S/RES/1820 (19. Juni 2008), S/RES/1888 (30. September 2009), S/RES/1889 (5. Oktober 2009), S/RES/2106 (24. Juni 2013), S/RES/2122 (18. Oktober 2013), S/RES/2242 (13. Oktober 2015), S/RES/2467 (23. April 2019), S/RES/2493 (29. Oktober 2019). Siehe zur globalen Agenda unter anderem: Davies und True (2019); Gizelis und Olson (2016); Scheuermann und Zürn (2020).

6 Siehe bei Henley/Roy (2020), Garikipati und Kambhampati (2020).

7 Im Durchschnitt arbeiteten Frauen vor der Pandemie 4,1 h pro Tag unbezahlt, Männer hingegen 1,7 h.
